# Learning from the experience of maternity healthcare workers in Malawi: a qualitative study leading to ten low-cost recommendations to improve working lives and quality of care

**DOI:** 10.1186/s12884-018-1960-5

**Published:** 2018-08-17

**Authors:** Abi Merriel, Julia Hussein, Address Malata, Arri Coomarasamy, Michael Larkin

**Affiliations:** 1 Population Health Sciences, Bristol Medical School, University of Bristol, Department of Obstetrics and Gynaecology, The Chilterns, Southmead Hospital, Bristol, BS10 5NB UK; 2 Institute of Metabolism and Systems Research, University of Birmingham, Academic Department, Birmingham Women’s Hospital Foundation Trust, 3rd Floor, Metchley Park Road, Edgbaston, Birmingham, B15 2TG UK; 3 Independent Maternal Health Consultant, Fintray Gardens, Hatton of Fintray, Aberdeen, AB21 0HY UK; 4 Malawi University of Science and Technology, PO Box 5196, Limbe, Malawi; 5 School of Psychology, Aston University, Birmingham, B4 7ET UK

**Keywords:** Human resources for health, Maternity care, Health systems, Resource poor, Quality of care, Qualitative, Interpretative phenomenological analysis

## Abstract

**Background:**

In Malawi there are too few maternity healthcare workers to enable delivery of high quality care to women. These staff are often overworked and have low job satisfaction. Skilled maternity healthcare workers are essential to improve outcomes for mothers and babies. This study focuses on understanding the working life experience of maternity staff at district hospitals in Malawi with the aim of developing relevant low-cost solutions to improve working life.

**Methods:**

A qualitative study using semi-structured interviews was undertaken in three district hospitals around Malawi’s Capital city. Thirty-one staff formed a convenience sample, purposively selected to cover each cadre. Interviews were recorded, transcribed and then analysed using Interpretative Phenomenological Analysis complemented by Template Analysis to elicit the experience of maternity staff.

**Results:**

Staff describe a system where respect, praise and support is lacking. Many want to develop their skills, however, there are barriers to advancement. Despite this, staff are motivated; they are passionate, committed professionals who endeavor to treat patients well, despite having few resources. Their ‘superdiverse’ background and experience helps them build resilience and strive to provide ‘*total care’*.

**Conclusions:**

Improving working lives can improve the care women receive. However, this requires appropriate health policy and investment of resources. There are some inter-relational aspects that can be improved with little cost, which form the ten recommendations of this paper. These improvements in working life center around individual staff (respecting each other, appreciating each other, being available when needed, performing systematic clinical assessments and communicating clearly), leadership (supportive supervision and leading by example) and the system (transparent training selection, training being need driven, clinical skills being considered in rotation of staff). To improve working lives in this way will require commitment to change throughout the health system. Thus, it could help address preventable maternal and newborn deaths.

**Electronic supplementary material:**

The online version of this article (10.1186/s12884-018-1960-5) contains supplementary material, which is available to authorized users.

## Background

The health system and the workers within it are essential for enabling good maternal health [1]. However, there is a shortage of healthcare workers (HCWs) in the places that need them most. Despite this issue gaining increasing attention over the last decade [2], the Global Health Workforce Alliance estimates a shortfall of 12.9 million skilled HCWs by 2035 [3].

Skilled providers reduce maternal mortality [4]. The World Health Organization (WHO) predicts that good quality care at delivery could reduce the 300,000 annual maternal deaths by over one-third, the number of stillbirths by over 500,000 and neonatal deaths by 1.3million [1, 5].

Skilled providers are essential for care at delivery. Many countries with high maternal mortality rates fall below recommended staffing levels [2]. Insufficient numbers of HCWs affects both the quality of care for women, and the workload of other staff. Providers are overworked, have less opportunity for interaction with colleagues and consequently face reduced job satisfaction [6]. A focus on the working life experience of HCWs may identify locally appropriate strategies to improve working environments for staff, thus enabling an improvement in their performance [7].

The Malawian government have been addressing this issue for a decade [8], but the improvements that have been made are fragile [9]. A recent service provision assessment, revealed that a skilled birth attendant was available 24 h a day in fewer than 90% of hospitals, and only one-third of clinics [10]. This undoubtedly contributes to the high maternal mortality ratio in Malawi of 510 per 100,000 live births [11].

This study aims to understand the working life experience of HCWs at district level facilities in Malawi. It will illuminate the positive elements of their working lives, the challenges they face and enable relevant solutions to be developed.

## Methods

### Study design

This qualitative study used one-to-one interviews with HCWs and combined two approaches to data analysis to allow a powerful picture of experience to be formed. Interpretative Phenomenological Analysis (IPA) harnesses the lived-experience in an in-depth, bottom-up approach using solely the data from participants. This approach was employed to identify core themes in an intensively-analysed sub-sample of the interviews [12]. Template Analysis(TA) [13] is a top-down approach, which was used to extend and develop these themes across the remainder of the dataset. This combination has been employed in previous studies [14] and benefits from the combined strengths of each approach. TA complements IPA as a flexible means of developing and transferring the coding structure within a larger sample [13, 15].

### Context

The study took place near Malawi’s Capital. Participants were recruited from three government hospitals; a district referral center, a district hospital and a community hospital. The district referral center had approximately 15,000 deliveries annually. Care was delivered by consultant and trainee obstetricians, general practitioners, clinical officers, degree level or registered nurses, diploma level nurse midwife technicians, trained nursing auxiliaries and untrained patient and hospital attendants. The district hospital, with approximately 3,700 deliveries annually, had no doctors who deliver obstetric care but had the other cadres. The community hospital with approximately 4,700 deliveries per year had no doctors or nursing auxiliaries.

### Sampling

IPA requires participants to have a shared experience, to enable exploration of the common or conflicting ideas within and between cases [16]. Here, the common perspective was working in a government hospital in Malawi. A sampling technique of convenience was used to access those staff who were available when the researcher was present. This was complemented by a purposive approach to ensure different cadres of staff were represented. A sample of six to nine participants was desired for the IPA element of the study as this is the volume of cases for which we felt in-depth experiential analysis was feasible. Beyond that, we wanted to gain a broader perspective of the range of staff and also allow staff who wanted to participate to share their stories. We determined that approximately 10 interviews per site would allow both of these goals to be met.

### Data collection

Following ethical approval from the Universities of Malawi and Birmingham, HCWs of all cadres were invited to participate in the study. After obtaining written informed consent, interviews were arranged with staff at a time convenient to them and a unique identifier (pseudonym) allocated. Semi-structured interviews lasting 30–90 min were carried out using a topic guide (Additional file 1), recorded and then transcribed. Participants were invited to receive their transcript, and several requested this, although only one made minor alterations.

### Data analysis

IPA required a detailed analysis of a small number of cases [12]. Nine cases, with the richest experiential data, spread across sites and cadres of staff were selected. These transcripts were read, re-read, then coded by hand by AMe and in part by ML. Coding focused upon capturing the meanings of important work-related experiences, from the respondents’ perspective. The research team then reviewed the emerging themes and feedback was sought from participants. These themes formed a ‘template’ for the second phase of analysis. This was carried out independently of the IPA, allowing the analysis to be grounded in the lived experience, but also to cope with the volume of data collected. This template was then applied to nine interviews using the qualitative software NVIVO version 10. The sub-themes were modified to incorporate new ideas, before being applied to the remaining dataset. During the application to the remaining dataset, no further themes were added.

Each coded theme was explored further. The data was analysed by understanding the distribution of codes across the data. The relationships between themes were then explored. This was carried out by drawing out key ideas from each case and creating individual ‘maps’ of the key themes. Ideas that corroborated or were polarised were identified and considered across cases in addition to within cases this provided the opportunity to develop the contents within each theme more fully [17, 18].

### The position of the researchers

AMe undertook the interviews and analysis. As a medical doctor with a background in obstetrics and gynaecology this PhD student based in the UK, brought a clinical perspective to the analysis. ML provided supervision and triangulation on the developing analysis from the perspective of phenomenological psychology.

## Results

Interviews were carried out with 31 HCWs at three sites (Table 1). An overview of the thematic structure is presented in Table 2. It is important to understand the context of work for these HCWs (Table 3). They described difficult working conditions with too few staff, resources, low pay and poor facilities with an increasing number of patients.

**Table 1:** Interview participants by site, cadre and sex

	District Referral Hospital		District Hospital		Community Hospital	
	Male	Female	Male	Female	Male	Female
Patient attendant	–	–	–	1	–	–
Nursing Auxiliary	–	1	–	1	–	–
Nurse midwife technician	1	1	–	4	–	5
Nursing Officer	2	1	–	2	1	1
Clinical Officer	4	–	1	2	2	–
Doctor	–	–	–	1	–	–

**Table 2:** The thematic structure of the initial IPA and final IPA/TA hybrid analysis indicating the super-ordinate and minor themes in addition to the number of participants contributing to each super-ordinate theme

Initial IPA Template	Final IPA/TA Template (number of participants contributing to evidence for theme)		
**Support to do the job** Calling for help from clinicians Conflict between cadres Coping Strategies Feedback from colleagues Teamwork Inadequate facilities Leadership Management Night time worst Common goals Communication No control Nurses with the patients Resources Senior support Supervision Referral Food Important ᅟ **Being a healthcare worker is hectic but good outcomes are enjoyable** Clear responsibilities Enjoy job Pressure of work high Outside of work Quiet times Motivation for doing job Feedback from patients ᅟ **Treating all patients well; Physically, socially and spiritually.** Quality Improvement Want to do good Whole person care Respectful Care Caring about patients Not properly doing duties ᅟ **Continuing development to increase independence and recognition** Experienced worker Career history Hierarchical system Learning from experience Learning from experienced colleagues Learning from other cadres Personal ambition and achievement On the job training Role Models School hoping to go Motivation for becoming hcw Picked for school ᅟ **Incentives motivate, meet needs and encourage implementation** Absent from work Training Incentives embedded in the system Pay	**Wanting a culture of respect, support and praise: Systemic issues (31)**		
	Absent from work Training	Calling for help from clinicians	Feedback from colleagues
	Communication	Conflict between cadres	Clear responsibilities
	Good working environment	Hierarchical system	Inadequate facilities
	Incentives Embedded	Leadership	Management
	Food Important	Night time worst	No control
	Not properly doing duties	Nurses with the patients	Patients agency
	Pay	Poor clinical assessment	School hoping to go
	Pressure of work high	Quiet times	Referral
	Resources	Selection for training	Senior support
	Supervision		
	**Passionately, determinedly fulfilling a dream: Staff Motivations (31)**		
	Career history	Caring about patients	Common goals
	Conflict between cadres	Enjoying	Experienced worker
	Feedback from colleagues	Feedback from patients	Good working environment
	Teamwork	Hierarchical system	Inadequate facilities
	Personal ambition and achievement	Food Important	Motivation for becoming HCW
	Leadership	Responsibility	Outside of work
	Patients agency	Pay	Role models
	Senior support	Supervision	Training
	Want to do good job		
	**Treating patients well; Physically, socially and spiritually (31)**		
	Calling for help from clinicians	Caring about patients	Conflict between cadres
	Coping Strategies	Night time worst	Not properly doing duties
	Nurses with the patients	Poor clinical assessment	Pressure of work high
	Quality Improvement	Referral	Respectful Care
	Teamwork	Want to do good job	Whole person care
	**Continuing development to increase independence, recognition and prospects (31)**		
	Absent from work Training	Career History	Conflict between cadres
	Hierarchical system	Learning from experience	Food Important
	Learning from other cadres	Personal ambition and achievement	Learning from experienced colleagues
	On the job training	Pay	Picked for school
	School hoping to go	Selection for training	Training
	**Incentives motivate, meet needs and encourage implementation (30)**		
	Incentives embedded in the system	Training motivating incentives	Selection for training
	Pay	Food Important	
	**Commitment, communication and taking responsibility: Professionalism (31)**		
	Absent from work Personal	Caring about patients	Communication
	Conflict between cadres	Coping Strategies	Learning from experience
	Learning from other cadres	Learning from experienced colleagues	Responsibility
	Not properly doing duties	Other cultures	Teamwork
	Outside of work	Patients agency	Poor clinical assessment
	Quality Improvement	Respectful Care	Want to do good job
	Night time worst		
	**Superdiversity of healthcare staff (31)**		
	Career history	Experienced worker	Family background
	Learning from other cadres	Motivation for becoming HCW	Other cultures
	Patients agency	Outside of work

**Table 3:** Context of Working Environment in Malawian District Hospitals

Too few staff: *In maternity we are only six nurses. Six nurses to cover during the day, the same six nurses to cover during the night. So most of the times we work, we are, something like we are punished. (Ellen, Nurse midwife technician)* Not enough resources: *A crisis of no delivery packs, no forceps, wherever there is a tear you have to run to the theatre to check for the needle in order to repair the tear. So that is our nice working place. (Chiso, Nurse Midwife Technician)* Inadequate facilities: *The three beds assisting 400 women [in a month]. It’s, it’s, it’s a lot and ee I feel so bad because the space is so small and then, some of patients may be delivering on the floor. (Kingston, Nursing officer, incharge)* Increasing workload: *The president announced that no one should be delivering to them [traditional birth attendants] so more are coming. And when they deliver at home they give penalty to the chief so in fear of giving penalty to the chief so they are forced to come to the hospital. (Aubrey, Clinical officer)* Low pay: *The salaries are not enough to take us for thirty days.* (*Paul, Clinical officer)* Late pay for overtime: *You find that you work. Instead of going home to rest, you know this, there’s a shortage, let me cover the shortage at the end you agree that at the end you will have such amount they don’t give you. Then it’s an embarrassing and you are discouraged to work in extra hours, eh, which makes the people who are on normal duty to feel the work because for example yesterday, yester night I was alone (Alile, Nurse midwife technician)*	

### Wanting a culture of respect, praise and support: Systemic issues

Hierarchical relationships were a divisive but systemic feature of participants’ accounts of their interpersonal interactions. For example, Alile, a nurse midwife technician(NMWT), described a ‘*demarcation’* between staff that exists between and within cadres. Paul a clinical officer(CO) believed this hierarchy ‘*play some some role in um actually weakening the teams’.*

Staff felt inferior ‘*we don’t have any due[feel able to] to tell them what, what can we do’* and uncared for ‘*they don’t regard us they don’t look at our welfare’ (Alile NMWT).* Staff had insight into the effects of this hierarchy. Victor, CO, felt that ‘*sometimes I may not be humble enough to take their[nurses’] suggestions’.*

Climbing the hierarchy by upgrading often meant spending less time delivering care. The ward staff were left to work, whilst noting that their seniors and role models were largely absent from the ward. For example:

*‘Sometimes they can come in the morning, they just walk walk [through] then they go out…the in-charges…the matron eeh is so difficult…yeah, she come here but she don’t work. She just stay, then she go, she come at lunch eat, go.’(Rhoda, NMWT).*

This lack of ward leadership meant that staff were frustrated, demoralised and had few opportunities to learn from senior, experienced colleagues. They valued being able to consult their senior colleagues. However, in its current format, HCWs felt that supervision was problem focused rather than supportive:

*‘They usually will always come to ask for something or to probe more if a problem comes in. That’s when they would like to know more but on the good things that we do, no.’ (Cynthia, Nursing Officer (NO))*

The lack of support extends to being transferred, a situation in which staff feel that they have no control. For example. Alile struggled to cope with the idea of being transferred because, ‘*they can…remove me from here to work somewhere regardless of my feelings and how can I cope with the other ward’.* In addition to this being personally challenging, it could leave wards with inexperienced staff: ‘*as for maternity we don’t have lots lots of those experienced ones it’s just a youthful generation’ (Kennedy, NO).*

### Passionately, determinedly fulfilling a dream: Staff motivation

Many HCWs felt that ‘*the dream I had, I have fulfilled’ (Ellen, NMWT).* Victor a CO described a common motivation of helping people: ‘*I wanted to be…one of the people that could be helping other people’.*

HCW motivation could be enhanced when staff appreciated each other as Roshin, a NMWT described: *‘even just appreciating, oh you are working hard, you are a hard worker you feel better’*. However, patients approaching staff could provide more powerful feedback:

*‘I feel motivated because of like the feedback that I get from people yeah cos [because] I could meet some people maybe a mother and her baby…she will come to me and say this is your child you delivered me during that time so I feel like wow this is great.’ (Vincent, NO)*

Staff felt recognised, respected and appreciated when approached by patients. As with Vincent, they spoke of these experiences fondly and found them motivating.

### Commitment, communication and taking responsibility: Professionalism

HCWs identified as professionals and wanted to do a good job. For example, when there was a staff shortage, the ward in-charge showed commitment and came in to ‘*make sure that care of the patient is not compromised’ (Kingston, NO, in-charge).* Kingston later reflected that ‘*there’s a lot of things in our facility that are supposed to be changed’*, this showed an understanding of the need to make changes, perhaps the first step in taking responsibility.

Similarly, other staff also tried to ‘*improve on healthcare where maybe something went wrong’ (Ash, CO).* Ash described the importance of reflecting together in a constructive rather than critical way:

*‘The goodness that it [reflecting constructively] doesn’t pinpoint on the fingers, that you are the one who did this thing. No but it’s like a general consensus, agreeing on what went wrong in the management of the patient.’*

Staff shared stories of how they reflected on their professional practice. One CO, Aubrey, described how he was spurred to do this after an incident where a woman died because of poor communication:

*‘So, he did spinal anaesthesia and I think it went high, then when I was cutting the abdomen, I saw dark blood, but I didn’t communicate to the anesthetist that the patient’s blood is deoxygenated, and the patient died.’*

He explained how this experience *‘touched me’* and how *‘since then I do communicate’*. However, not all staff had such negative experiences informing their professionalism. For example, Leoni, an NO incharge, shared a positive experience, when she addressed lack of professionalism openly:

*‘I learned that if you communicate with your people properly, things will actually run smoothly. Because, in the beginning, I saw that people would give so many excuses for not coming to work. That was when we were all new in this ward. Then we had a meeting. We sat down, they told me their problems, we sorted it out. And now when someone gives me an excuse I know that it’s genuine cause you can see that there are less absenteeism’s.’ (Leoni, NO)*

As Leoni highlighted, good quality respectful communication enabled problems to be solved within the team. This idea could be invaluable as staff described strained relationships when colleagues behaved unprofessionally. As the matron Violet reported, the COs *‘get missing’* after ward rounds. Perhaps it is because ‘*they go to another department but most of the time they are out of the hospital’.* This meant that ‘*when you have got a an emergency, or you have got a very sick patient you have to search for them’.* Violet’s sentiments were echoed universally by the nursing staff. Chiso, a NMWT, felt *‘that’s how the bad teamwork comes with the clinicians’.* He described how relationships could become strained when nurses call the clinicians: ‘*we jot down…how we called the clinician[CO] he said he is coming…we called him again he did not come; so they hate those things.’*

However, Kaia, a patient attendant, described how people working together *‘can’t agree on everything’* but they needed to behave in a professional manner and *‘concentrate on the positive things and meeting the goal for our jobs’.*

### Treating patients well; physically, psychologically, spiritually

Theresa, NMWT, described how as a professional ‘*you need to care psychologically, socially, physically….spiritually’* for your patients. This meant providing high quality, respectful care, which could be challenging especially when ‘*the women sometimes they irritate you’ (Alile, NMWT).* Natasha, a CO, recognised that this was challenging for some staff because ‘*they will come with their own problems at home’* but still she doesn’t *‘think you can just come here and start shouting at everyone it’s not on’.* Alile described how instead ‘*you just make your heart to calm’*.

Conflict between cadres impacted on quality of care for patients who, according to Francis a CO ‘*overstay at the hospital’* due to ‘*our absence’* because *‘there is no one to work on them’.* This forced staff to work outside their zone of competence, resulting in increased workload and poorer patient care:

‘*Patients rush to the wards. Can you please assist us, the clinician is not there. So we are the ones may be admitting the patients, prescribing drugs…clinicians are the ones who know more about drugs than a nurse.’ (Rachel, NMWT).*

Perhaps because the *‘clinician [CO]…is not coming’(Brenda, NMWT),* nurses needed to make clinical decisions. Victor, CO, described how he operated on patients without assessing them because the patient ‘*is already there’.* To compound this issue, there was delay, because nurses believed they were more skilled than clinicians and so called for help only when ‘*maybe we have failed that’ (Vincent NO)*.

### Continuing development to increase independence, recognition and prospects

Many HCWs like Kingston, NO, felt they *‘need to go back to school to further my education’.* This motivation could have been driven by a desire to help patients, but in many cases, upgrading provided the opportunity to improve job prospects: ‘*there are better salaries than what I am getting here now’ (Rhoda, NMWT).*

Upgrading was not easy. Staff often have to go back to secondary school. Getting a first degree was still a *‘challenge’* but according to Marshall, a CO *‘after first degree the world is open here in Malawi’.* The competition for places to upgrade was fierce and many like, Alile, NMWT, felt that ‘*the chance is very low’.*

HCWs recognised that ‘*experience is a good teacher’ (Pricilla, NMWT).* They appreciated learning from each other and learning something from a lower cadre could be particularly memorable: ‘*suturing itself I was taught by a maid [patient attendant]…I still remember that suturing even though the patient attendant doesn’t know suturing but since they have been there for years’ (Natasha, CO).*

### Incentives motivate, meet needs and encourage implementation

In terms of continuing development training sessions were inextricably linked to attendance allowances as Rachel, NMWT, described: *‘It’s the same like when you train your child to eat breakfast every day before he goes to school he is needs to take breakfast…The other day when you are not going to prepare breakfast for that child that child won’t be happy…we got used already to get an allowance after a training.’*

HCWs wanted allowances because they *‘could have been at home, doing some other things that could have brought us some monies’ (Sasha, NMWT).* Training sessions often resulted in more work, implementing interventions. Ellen, NMWT, felt that without incentives ‘*they say I haven’t received anything I will work as I am supposed to work so they don’t even implement those things’.*

There was a feeling of unfairness about training sessions, according to CO Aubrey, ‘*they don’t balance chances of attending meetings’.* Theresa, NMWT described how *‘most of the time they consider the registered ones so you are always on duty…so that makes us down’.* This could feed into staff becoming demoralised.

### Superdiversity of healthcare staff

Staff came from different perspectives and backgrounds. This multilayered complexity within the population of HCWs reflected superdiversity [19]. Some, like Cynthia, NO, were motivated by ‘*that feeling of helping others’,* whilst others were motivated by personal experience. For example, when Francis, CO, was being a guardian to his father he *‘wasn’t much convinced’* with ‘*the way they were attending…patients’.* For others, being a HCW is a job *‘I wanted to become an accountant that was my dream…upon been applying to University of Malawi and been left out I had no choice’ (Ash, CO).*

Staff also had a diverse range of prior experience. For example Francis ‘*was a teacher myself’.* Memory, NMWT, had a wealth of experience, having ‘*qualified in 1986’* and worked in ‘*different kinds of nursing’.* Violet, a matron, saw the importance of experience because staff bring *‘new ideas…how they were doing things…in their various hospitals’* which could *‘assist us to change’* and *‘learn new things’.*

Staff had different challenges at home, especially the women, ‘*the child is sick…we come here thinking how’s my kid at home so sometimes being a working mother…that’s a challenge’* (*Roshin, NMWT).*

### Positivity, teamwork and improvisation: Resilience

HCWs display resilience, an ability to cope with the difficult conditions in which they work. For some, this resilience is rooted in motivation and professionalism, or for others resilience is grounded in their self-belief. For example, Kennedy and NO, shows the vision that drives him: ‘*I always believe…I could be that person, that single person that could bring change’.*

Some practical things helped staff in their working lives. Marshall, CO, described how ‘*you should do cover’* when ‘*my friend has going out’*. Whilst the negative aspects of absenteeism are undeniable, having supportive workmates to rely on when you needed to *‘it really helps’.* Cynthia, NO, described, how the incharge’s put ‘*experienced people and working with inexperienced’* so that the ward *‘does not want’.*

Cynthia described another important coping mechanism, teamwork, whereby ‘*all the resources are put to where it’s busy’* so once ‘*they’ve finished doing their work in postnatal ward, they have to assist in labour ward’.* This idea of building resilience by learning from experienced staff is described by Yvonne a doctor. She takes the opportunity to learn skills from a consultant, so she can be more resilient as a doctor, coping alone in future:

*I always try to not be a superhero…I would rather a consultant watch...next time I’m faced with that situation I know exactly what to do.*

Personal resilience could be built, for example by diversifying income as described by Natasha, CO, ‘*I have a shop in town*’. Whilst some have extra jobs that resulted in absenteeism, others like Natasha were able to manage their extra earning around work *‘most of the time is her who takes care I just go there for stock taking and everything’.*

Family builds resilience as staff have to *‘actually find some time to go visit the family’(Paul CO).* Staff could also ensure that they coped by asking ‘*for an off to rest’ (Rhoda, NMWT).* The general ebb and flow of clinical work could help as sometimes it was quiet *‘yesterday we only had a single patient, so I was just seated there’.*

Finally, HCWs *‘are socially people who are respected* so *being a nurse, it’s good’(Kingston NO).* This positive reception, bolstered staff morale and built their resilience.

## Discussion

Maternity HCWs in Malawi are delivering care within a challenging environment and whilst navigating complex interpersonal relationships. These factors make work life challenging and erode the ability to provide excellent patient care. Despite this, staff seemed intrinsically motivated and draw on their resilience as a person and a team to care for their patients.

### Strengths and limitations

A strength of this study is that it provides insight into a cross section of district level hospitals in Malawi. The complementary IPA and TA approaches allow an in depth understanding of the data whilst incorporating the breadth of responses.

Whilst these facilities were based only in two districts, and with a limited number of staff, staff of all cadres were invited to share their views. However, due to time and resource constraints, no ward clerks and only a few doctors and auxiliaries participated in the interviews. Having said this, when conducting the template analysis for the final 13 interviews, no new themes emerged. This may suggest that our sample did enable us to gain a good picture of the experience of healthcare workers.

Despite this small sample size, the findings of the study are in-keeping with similar studies in low-resource countries. This may mean that the findings could be considered useful in other comparable settings.

### Interpretation

**Fig. 1:**
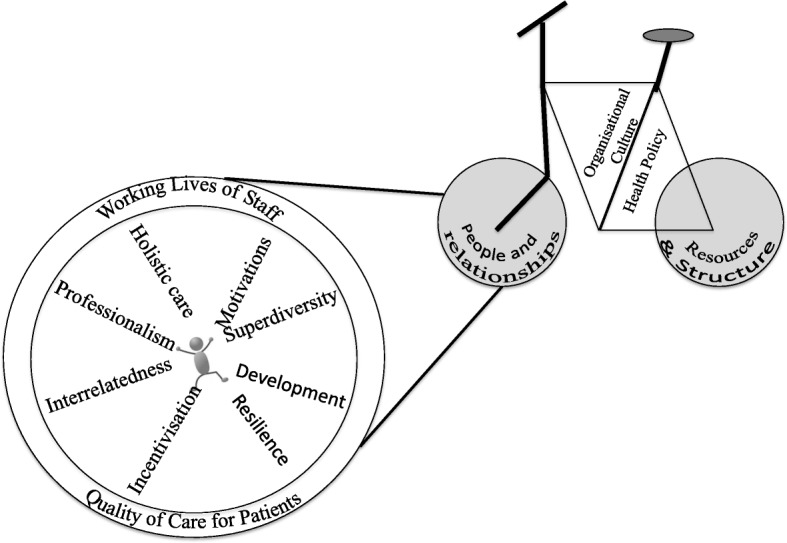
The Health Systems Bicycle with the people and relationships wheel enlarged

Well-functioning interpersonal relationships are vital to delivering care, however, a functioning health system also requires thoughtful health policy and resources. This is illustrated using the health systems bicycle (Fig. 1).

For the health system to travel towards high quality care all components must function well. When considering the inter-relational aspects within the health system, each ‘spoke’ is important to ensuring the wheel can keep turning, as is each element identified in this study important for HCWs. Learning from each element and developing positive solutions to strengthen each ‘spoke’, can allow for better support for working lives and patient care. Unlike the ‘health resources’ wheel, many of the solutions to improve interrelatedness require no or few additional resources, but instead personal commitment from healthcare workers and a supportive framework of health policy. With this in mind ten recommendations have been developed.

These ten recommendations are grouped within the three domains of individual inter-relational issues, leadership and healthcare system (Table 4). The individual’s contribution to improving relationships at work form the first five recommendations:

1. Staff feel that they work in a hierarchical system which demoralises them as individuals and weakens teams, treating each other with greater respect could go some way to improving this.
2. Further motivation could be fostered by openly appreciating colleagues.
3. Being available when other members of staff need you may be more challenging, as HCWs are operating within difficult personal circumstances too, but it would improve relationships between staff and care for patients.
4. Once with a patient, performing a systematic clinical assessment can improve clinical decision-making.
5. Communicating clearly can improve patient outcomes and increase the likelihood of a satisfying good outcome.

**Table 4:** Ten low-cost recommendations to imporve working life for maternity healthcare workers and quality of care for women

Recommendation	Evidence from the data	Studies with related findings
**All Staff Should**….		
1: …show respect towards all colleagues regardless of their educational background and gender	*the qualifications also play some some role in um actually weakening the teams. Because people think ahh why should I be taking knowl, i mean ideas from you, why should I be taking decisions from him? I think I’m more superior in terms of qualification.* Paul, Clinical officer	Chimwaza 2014 [22]
2: …express appreciation to their colleagues of all cadres	*if he sees that you have done something great on the patient, he says you’ve managed patients very well. He used to say that…It makes me feel happy. You feel encouraged that I should do even more than this.* Fanny, Clinical officer	Mathauer 2006 [28]
3: …be available to perform clinical duties as per their job role	*I think its being selfish yea because sometimes when they are on call let’s say during the night, you phone them, they can’t even res respond to their phones.* Rachel, Nurse Midwife technician	Chodzaza 2010 [20]
4: …perform systematic clinical assessments when indicated	*maybe you want to examine the patient thoroughly but say ah am tired. Just take out your tongue I just want to see if you are pale or not. Ah she’s fine and you continue writing something instead of doing thorough examination* Fanny, Clinical officer	
5: …communicate clearly about clinical issues to each other	*they don’t allow to go together to see patients so they just let you to go alone. Writing your plans in the files of patients. Then they just do it what you have written so come tomorrow the same thing, nothing has happened. So you again write may be take, check a full blood count. Come tomorrow, no sample taken so that’s what happens* Aubrey, Clinical officer	Bhattacharyya 2015 [30]
**Leaders should**…		
6: …take a supportive approach to all education and supervision with a focus on highlighting the positives and providing constructive criticism	*if the management would be flat out encouraging and if in like there’s this like with performance appraisals if you have done, done a good job you get appraised, not with money, but just maybe just somebody patting you at the back’ well done’ you continue. Or ok you have done better but what if you’d do it this way next time so you’ll be much more better than this. I think that kind of comment, of spirit is lacking in the management.* Victor, Clinical officer	Mathauer 2006 [28] Bradley 2009 [21]
7: …lead by example	*They[leaders] are not supposed to be staying in the office the whole week, the whole month without going to the ward and seeing what’s happening there.* Cynthia, Nursing officer	Mathauer 2006 [28]
**The system should**…		
8:…have a transparent and fair process for selection to upgrade or attend training sessions/workshops/seminars/	*when it’s saying that everyone, eve every nurse should be trained, we do. But it’s when they say we need only two nurses to be trained on this, then there is also favoritisms.* Alile, Nurse midwife technician	Songstad 2011 [31] Manafa 2009 [24] Mathauer 2006 [28] Wurie 2016 [25] Chimwanza 2014 [22]
9: …ensure that any training offered to staff is driven by the skills and knowledge staff need and desire, rather than attendance being motivated only by the incentives	*I was supposed to be to train people for tubal ligation and other family planning methods. And they were booked in a hotel they said everything is being paid for what they will be given back is their transport so the same day the people said we cannot just be staying here, this is like away from home its like we are having now two houses we have to manage our home there we are here so they said not giving you are not going to give us anything it will not be possible so the training was cancelled the same day everybody goes back.* Natasha, Clinical officer, incharge	Manafa 2009 [24] Mathauer 2006 [28]
10: …carry out rotations or relocations of staff according to clinical need rather than any other agenda	*like currently the the labour ward has no HTC[HIV testing and counselling] provider and the all the nurses all of us that are working there we are not trained in doing HIV testing and counselling…yeah so, it’s always a challenge, there are some women which we are missing yeah…it’s almost a year now…they are usually a dispersed to other departments during the time they are doing the rotation* Vincent, Nursing officer, incharge	

The next two recommendations require leaders to contribute to make a positive difference to relationships at work:

(6) Leaders need to take a motivational, rather than belittling approach to supervising staff and perhaps most importantly,
(7) Leaders should lead by example. By leaders avoiding clinical work staff cannot be blamed for wanting to escape clinical work. A generation of excellent clinical leaders could provide a foundation for positive change within the health system.

The final three recommendations need to occur at the level of the health system:

(8) Perceived unfairness in selection for training and further education demoralises staff. Creating a transparent and fair system for selection could alleviate some of these feelings.
(9) As illustrated in the incentives theme, staff value allowances more than the training, ensuring training is targeted to staff needs could begin to address this issue. For this to change staff, the government and donors will need to develop an open dialogue.
(10) When staff are relocated in the regular re-assignments that occur, the clinical needs of the hospital and district must be a priority but staff’s personal needs should also be considered. HCWs are not generic, they have different specialist skills and experience. Appreciating this could improve the skill-mix of staff enabling better patient care and senior support.

Additional evidence for each of these ten recommendations is presented in Table 4 where you can see that many of these observations are not new or confined to Malawi. However, to our knowledge these strategies have not been collected as a suite of low-cost recommendations. For example, studies from Malawi [20–24] and elsewhere [25–28] show that HCWs would like access to training opportunities, supportive supervision and appreciation from their colleagues.

## Conclusions

This study has provided an insight into the working lives of maternity healthcare workers in Malawi at an important time. The international maternal health community is shifting its focus onto the Global Strategy for Women, Children and Adolescents. The ten low-cost strategies identified in this study could be used to address two of the strategic priorities for ending preventable maternal and newborn mortality and stillbirths: strengthening care around the time of birth and strengthening health systems [29].

## Additional File

Additional file 1: Topic Guide. Topic guide and prompts to understand working life experience of healthcare workers in Malawi. (DOCX 12 kb)

## Abbreviations

CO: Clinical officer
HCWs: Health care workers
IPA: Interpretative phenomenological analysis
NMWT: Nurse midwife technician
NO: Nursing officer
TA: Template analysis
WHO: World Health Organization
