# Supplementary material for: Learning from the experience of maternity healthcare workers in Malawi: a qualitative study leading to ten low-cost recommendations to improve working lives and quality of care
Source: BMC Pregnancy Childbirth. 2018 Aug 17;18:336. doi: 10.1186/s12884-018-1960-5 (PMC6098626; doi:10.1186/s12884-018-1960-5)
Supplement: Supplementary file 1 — Topic Guide. Topic guide and prompts to understand working life experience of healthcare workers in Malawi. (DOCX 12 kb) [file 12884_2018_1960_MOESM1_ESM.docx]

### Topic guide for working life individual interviews

**Understanding the working life experience of healthcare workers in Malawi.**

Please could you describe what you do at work?

Prompts: What happens typically? How do you cope? How do you feel? What are your responsibilities? What are your skills?

How did you come to be a (doctor/clinical officer/nurse)

Probe: how do you feel about that now?

If a new post came up at this facility, what would you say to a prospective applicant about working here?

Prompts: What tasks need to be performed? How do co-workers interact? What is the physical environment like?

Tell me about the support you get to fulfil your role.

Prompts: Is there someone you can call for help? Are there other team members around? How does that make you feel?

Tell me about your continuing professional development?

Prompts: Can you attend? Why do you attend? How does that help you?

What are your long-term career plans?

Prompts: continue working here? Leave to another healthcare job? Leave healthcare?

How does your day at work impact upon your home life?

Prompts: how does your working schedule fit around your home life- predictable/unpredictable? Does the workload mean you’re tired at home? Does the work you do energise you?

How do you feel the staff at the centre work together?

Prompts: how does the team work in an emergency? Does the team get on well? Do the nurses/doctors/clinical officers/managers/assistants communicate well between groups? Are there any things that are particularly good about the team? Are there any problems within the team?

Do you think that your working life could be made better?

Prompts: Teamwork? Training? Systems? How might that work? How could you achieve that?
